# Expression and Antagonistic Activity Against Plant Pathogens of the Phage Tail-like Protein from *Burkholderia multivorans* WS-FJ9

**DOI:** 10.3390/microorganisms13040853

**Published:** 2025-04-09

**Authors:** Tong-Yue Wen, Xing-Li Xie, Wei-Liang Kong, Xiao-Qin Wu

**Affiliations:** 1 Co-Innovation Center for Sustainable Forestry in Southern China, College of Forestry, Nanjing Forestry University, Nanjing 210037, China; 2 Jiangsu Key Laboratory for Prevention and Management of Invasive Species, Nanjing Forestry University, Nanjing 210037, China

**Keywords:** *Burkholderia multivorans*, phage tail-like protein, prokaryotic expression, *Phytophthora cinnamomi*

## Abstract

Microorganisms exert antagonistic effects on pathogens through different mechanisms, thereby achieving biological control of plant diseases. Many *Burkholderia* strains can produce complex secondary metabolites and substances that have toxic effects on host cells. The phage tail-like bacteriocins (tailocins) is a compound with antibacterial activity. However, its function in *B. multivorans* has not yet been reported. This article explores the ability of *B. multivorans* WS-FJ9 to antagonise plant pathogenic fungi and oomycetes, screening the potential tailocins in the strain WS-FJ9 and verifying their function, to reveal its novel antimicrobial mechanisms. We found that WS-FJ9 had strong antagonistic effects on the plant pathogenic fungi *Phomopsis macrospore* and *Sphaeropsis sapinea*, and the pathogenic oomycete *Phytophthora cinnamomi*. The phage tail-like protein Bm_67459 was predicted from the WS-FJ9 strain genome. The Bm_67459 cDNA encoded 111 amino acid sequence, and the relative molecular weight was approximately 11.69 kDa, the theoretical isoelectric point (pI) was 5.49, and it was a hydrophilic protein. Bm_67459 had no transmembrane helix region or signal peptide, and it belonged to the Phage_TAC_7 super family. qRT-PCR results showed that *Bm_67459* gene expression was significantly upregulated during contact between WS-FJ9 and *P. cinnamomi*. The purified Bm_67459 protein significantly inhibited *P. cinnamomi* mycelial growth at 10 μg·mL^−1^. In summary, the WS-FJ9 strain had broad-spectrum anti-phytopathogenic activity, and the tailocin Bm_67459 was an important effector against the plant pathogen *P. cinnamomi*, which helps to reveal the antagonistic mechanism of this strain at the molecular level and provides excellent strain resources for the biological control of plant diseases.

## 1. Introduction

Plant diseases have always been an important factor threatening agricultural and forestry production. To control plant diseases, farmers usually apply methods that include chemical control, physical control, and biocontrol [1]. Chemical fungicides are expensive, toxic, and hazardous with many environmental problems. Finding safe and nontoxic biocontrol bacteria to control plant diseases has become a research hotspot in recent years [2]. The mechanisms of biological control include mainly competition, plant induced systemic resistance (ISR), and antagonism [3]. Among them, antagonism is the most important antibacterial mechanism. Biocontrol bacteria can produce antibacterial proteins, antibiotics, and bacteriocins to inhibit plant pathogenic microorganisms [4]. Bacteriocins are potent antimicrobial peptides that are produced by bacteria, with extensively post-translationally modified structures encoded on complex gene clusters. Bacteriocins are ribosomally synthesised peptides secreted by a variety of bacteria for the purpose of killing other bacteria, and they participate in removing microbial competition in prokaryotes [5,6]. Additionally, some phage tail genes can express proteins with bacteriocin-like functions, i.e., phage tail-like bacteriocins (tailocins). Tailocins are widely distributed in Gram-negative bacteria and exhibit strong specificity towards their targets, greatly affecting the surrounding microbial community [7]. In recent years, studies have found that this type of protein has antagonistic activity against plant pathogens [8].

Tailocins derived from prophages are a unique class of bacteriocins that are composed of many different polypeptide subunits [9]. F- and R-type pyocin-encoding gene clusters of *Pseudomonas aeruginosa* PAO1 are almost identical to the tail genes of the λ and P2 phages, respectively [10]. Nearly 65% of completely sequenced bacterial genomes contain prophage genes, and some bacteria carry prophage sequences that account for 20% of the capacity of the bacterial genome [11]. Prophages integrations confer virulence factors to bacteria and enhance bacterial adaptation to the environment [12]. This type of protein can have toxic effects on certain plant pathogenic bacteria and fungi. For example, the S-type pyocin produced by *Pseudomonas fluorescens* SF39a and the tailocin produced by strain SF4c have antibacterial activity against several plant pathogens of *Xanthomonas* and *Pseudomonas* [13,14,15,16]. Yao et al. found that the phage-like tail protein BceTMilo can antagonise 60% of *Burkholderia cepacia* complex (Bcc), representing 10 species and 90% of non-Bcc *Burkholderia* strains [17]. One kind of R-type pyocin can kill *Campylobacter*, *Neisseria,* and *Haemophilus* [18]. However, at present, studies of tailocins are focused mostly on inhibiting plant pathogenic bacteria, and there are still few reports on the inhibition of plant pathogenic fungi and oomycetes. Swain et al. found that *Burkholderia gladioli* NGJ1 secreted the prophage tail protein Bg_9562 through the type III secretion system, which had antagonistic effects on plant pathogenic fungi and oomycetes such as *Rhizoctonia solani*, *Fusarium oxysporum*, *Magnaporthe oryzae,* and *Phytophthora* sp. [19]. However, it has not been reported whether there are similar phage tail-like proteins in other *Burkholderia* strains that can antagonise plant pathogenic fungi and oomycetes.

The plant growth-promoting bacterium *B. multivorans* WS-FJ9 was screened from the rhizosphere of pine trees in our laboratory [20]. Previous research has shown that WS-FJ9 effectively promoted plant growth and dissolved phosphate, and had a certain antagonistic effect on plant pathogens [21,22], but the specific antagonistic mechanism was still unclear. Liu et al. reported genome data for *B. multivorans* WS-FJ9 and analysed and verified the phosphate-dissolving-related genes of this bacteria [23]. This study explored its antagonistic effects and mechanism against a variety of forest pathogens. At the same time, whole-genome data of WS-FJ9 was analysed, the sequences were blasted to find phage tail-like protein genes with potential antibacterial function, and a protein encoded by such a gene was prokaryotically expressed and purified. The possible role of this gene was explored in the antagonism of the WS-FJ9 against plant pathogens. These results were expected to lay a foundation for the in-depth study of the role and function of this protein and other prophage tail proteins in *Burkholderia* and provide new ideas for the prevention and control of plant diseases.

## 2. Materials and Methods

### 2.1. Bacteria and Plant Pathogens

The plant growth-promoting bacteria, *B. multivorans* WS-FJ9, was isolated from the rhizosphere soil of a slash pine (*Pinus elliottii*) in the Guangzhuang Forestry Center, Fujian, China [20], and deposited in the Chinese Center for Type Culture Collection (Accession No. CCTCCM2011435). Plant pathogens: *P. cinnamomi* (causes cedar root rot), *R. solani* (causes pine seedling damping-off), *Phomopsis macrospore* (causes poplar canker), and *Sphaeropsis sapinea* (causes pine shoot blight) were stored in the Laboratory of Forest Pathology, Nanjing Forestry University.

### 2.2. In Vitro Antifungal Activity

We used a 0.5 cm hole punch to take plugs containing mycelia from the aforementioned 4 plant pathogens, which had been cultured on a potato dextrose agar (PDA) plate (containing 20 g of agar, 20 g of dextrose, 200 g of potato, and 1 L of sterile water) for 7 days. Then, each pathogen was inoculated on an inverted PDA plate with a sterile needle by the direct contact method. Each plug was added to 25 μL of WS-FJ9 fermentation broth and cultured for 3 days in Luria–Bertani (LB) medium (containing 5 g of yeast extract, 10 g of tryptone, and 10 g of NaCl per litre, pH 7.0). Three plugs were placed in each empty culture dish, and three culture dishes were prepared in each treatment. The same amount of normal saline was added to each plug in the control group. After 72 h, mycelial growth of the pathogen was observed by a Zeiss SteREO Discovery.V20 microscope (Oberkochen, Germany).

### 2.3. Bioinformatics Analysis of the Phage Tail-like Protein Bm_67459 of B. multivorans WS-FJ9

According to the genome data of the WS-FJ9 [23], phage tail-like proteins were screened, and a homology analysis of selected protein sequences in the NCBI database was performed using BLAST (https://blast.ncbi.nlm.nih.gov/, accessed on 7 May 2024). It was found that contig67459 had high similarity with a reported prophage tail-like protein with antibacterial activity, Bg_9562 [19], so we renamed the gene *Bm_67459*. DNAMAN software (v10.0) was used to compare the amino acid sequences of the Bm_67459 and Bg_9562 proteins. ExPASy online analysis software was used to analyse the physical and chemical properties of the Bm_67459 protein (https://web.expasy.org/protparam/, accessed on 7 May 2024), and the transmembrane region of the Bm_67459 protein was predicted by the TMHMM Server v. 2.0 (http://www.cbs.dtu.dk/services/TMHMM/, accessed on 7 May 2024). The NetPhos 3.1 Server tool was used to analyse phosphorylation sites encoded by the *Bm_67459* gene (http://www.Cbs.Dtu.dk/services/NetPhos/, accessed on 7 May 2024), while SignalP-4.0 software was used to predict the N-terminal signal peptide sequence of the Bm_67459 protein (http://www.cbs.dtu.dk/services/SignalP-4.0/, accessed on 7 May 2024). In addition, the NCBI conserved domain database (CDD) was used to analyse the functional domain of the Bm_67459 protein (https://www.ncbi.nlm.nih.gov/Structure/cdd/wrpsb.cgi, accessed on 7 May 2024), and its secondary structure was analysed at the online software SOPMA (https://npsa-prabi.ibcp.fr/cgi-bin/npsa_automat.pl?page=npsa_sopma.html, accessed on 7 May 2024). Finally, SWISS-MODEL software (https://swissmodel.expasy.org/, accessed on 7 May 2024) was used to predict and analyse the three-dimensional structure of the Bm_67459 protein (http://swissmodel.expasy.org, accessed on 7 May 2024).

### 2.4. Gene Cloning of the Phage Tail-like Protein Bm_67459 of B. multivorans WS-FJ9

According to the whole genome sequencing and annotation results for the WS-FJ9 strain, specific primers of the phage tail-like protein gene *Bm_67459* were designed by Primer Premier 5.0 (Premier Biosoft International, Palo Alto, CA, USA), as shown in Table 1. Total DNA was isolated from WS-FJ9 cells using a SteadyPure Bacteria Genomic DNA Extraction Kit (Accurate Biology, Changsha, China), and then the *Bm_67459* gene was amplified. The PCR system used included 2 μL of DNA template, 2 μL of primer pairs, and 10 µL of Green Taq Mix, and it was adjusted to 20 μL in volume with ddH_2_O. The conditions for amplification were enzyme activation at 98 °C for 5 min, denaturation at 98 °C for 30 s, and hybridization at 65 °C for 30 s. The PCR products were detected by 1% agarose gel electrophoresis. Recovery and purification of PCR products was performed using a TaKaRa MiniBEST Agarose Gel DNA Extraction Kit Ver. 4.0 (TaKaRa, Dalian, China). The above purified PCR product was ligated to the pMD^TM^19-T cloning vector and transformed into competent *Escherichia coli* DH5α cells. Then, the cells were evenly spread on LB solid medium containing ampicillin (100 µg·mL^−1^) and cultured at 37 °C. Once transformed clones were identified, bacterial suspension from each clone was sequenced at Nanjing GenScript (Nanjing, China).

### 2.5. qRT-PCR

After adding a drop of the fermentation suspension of WS-FJ9 to the surface of pathogen plugs and incubating for 24 h, the surfaces of the plugs were repeatedly washed with 0.9% normal saline so that the bacteria were suspended in normal saline. The bacteria were centrifuged at 5000 r·min^−1^ for 5 min to complete the collection of WS-FJ9. A bacterial total RNA extraction kit was used to extract the total RNA of WS-FJ9 (TransGen Biotech, Beijing, China). cDNA was synthesised from the extracted bacterial RNA by HiScript II Q Select RT SuperMix for qPCR (Vazyme, Nanjing, China). Specific primers were designed by Primer 5.0 software for quantitative real-time PCR (qRT-PCR) (Table 1), and the 16S rDNA gene of WS-FJ9 (Accession No. KY003099.1) was chosen as a reference gene [18]. qRT-PCR was carried out in a 20 μL system that contained 2 μL of the diluted cDNA, 10 μL of AceQ Universal SYBR qPCR Master Mix, 0.4 μL of each primer (10 μM), and 7.2 μL of ddH_2_O. Relative expression levels (fold) were determined using ABI Prism 7500 software (Applied Biosystems, Foster City, CA, USA) and the 2^−ΔΔCt^ method [24]. qRT-PCR was conducted with three biological and technical replicates.

### 2.6. Construction of a Prokaryotic Expression Vector with the Phage Tail-like Protein Bm_67459 Gene

According to the sequence characteristics of the *Bm_67459* gene and the restriction endonuclease recognition sites of the expression vector pET32a, EcoRV restriction sites (underlined portions) were added to upstream and downstream primers for the target gene (designed with Primer 5.0): E-Bm_67459-F (5′–AAGGCCATGGCTGATATCATGACGAACCACGCTCAACACG–3′) and E- Bm_67459-R (5′–GAATTCGGATCCGATATCCGCAGACGGGGATGCCAT–3′). The *Bm_67459* gene was amplified using WS-FJ9 DNA as the template and the high-fidelity enzyme Phanta Max Super-Fidelity DNA Polymerase (Vazyme, Nanjing, China). The PCR system (50 mL) consisted of 1 µL of dNTP Mix, 2 µL of DNA template, 4 µL of the primers F/R, 1 µL of DNA polymerase, 25 µL of 2× Phanta Max Buffer, and 17 µL of ddH_2_O. The PCR conditions were the same as those in Section 2.4. The correct size of the PCR product was observed by 1% agarose gel electrophoresis, and the PCR product was purified. The purified *Bm_67459* gene fragment was inserted into the EcoRV site of the His-tag-containing plasmid pET32a to generate the pET32a-Bm_67459 expression vector, which was transformed into *E. coli* DH5α cells. These cells were plated on LB solid medium containing 100 µg·mL^−1^ ampicillin and incubated overnight at 37 °C. The positive clones were sequenced, and restriction enzyme digestion ensured that the correct expression plasmid was obtained through subcloning.

### 2.7. Induced Expression and Purification of the Phage Tail-like Protein Bm_67459 of B. Multivorans WS-FJ9

The successfully constructed pET32a-Bm_67459 plasmid was transformed into *E. coli* BL21 cells. The empty pET-32a vector as a control was also transformed into *E. coli* BL21 cells. We selected colonies on LB solid medium with 100 μg·mL^−1^ ampicillin. After colony PCR identification, *E. coli* BL21 cells containing the pET32a-Bm_67459 plasmid or pET-32a were inoculated into LB liquid medium containing 100 µg·mL^−1^ ampicillin and cultured overnight at 37 °C. The bacterial suspension was inoculated into LB liquid medium at a ratio of 1:100 and cultivated at 37 °C to an OD_600_ of 0.6–0.8. Then, we added 0.5 mmol·L^−1^ isopropyl-β-thiogalactopyranoside (IPTG) to the bacterial solution, which was incubated at 37 °C under constant shaking for 4 h to induce expression of the recombinant protein. The bacteria were collected by centrifugation at 8000 r·min^−1^ for 10 min at low temperature, suspended in an appropriate amount of phosphate-buffered saline (PBS) (10 mM, pH 7.2–7.4), and ultrasonically disrupted in an ice-water bath. After disruption, the supernatant was collected and centrifuged, and the pellet was suspended in PBS. The supernatant and the precipitate were collected for SDS-PAGE electrophoresis, and the expression of the target protein was analysed by staining with Coomassie brilliant blue. The Bm_67459 fusion protein was purified from the supernatant using a Ni-NTA-Sefinose column (Sangon Biotech, Shanghai, China).

Preliminary experiments showed that the target protein was soluble. Therefore, the protein was purified from the supernatant by Ni affinity chromatography. The Bm_67459 fusion protein was added to the Ni-NTA-Sefinose column and washed with 5 volumes of Ni-IDA binding buffer (300 mM NaCl, 50 mM Tris-HCl, pH 8.0) until the OD_280_ value of the effluent reached a baseline. Then, the target protein was eluted with Ni-NTA washing buffer with imidazole concentrations of 50, 100, 150, and 250 mmol·L^−1^ at a flow rate of 1 mL·min^−1^, and the effluent was collected. The abovementioned protein solution was poured into a dialysis bag, and PBS (0.2 g of KCl, 3.58 g of Na_2_HPO_4_.12H_2_O, 0.27 g of KH_2_PO_4_, 8.8 g of NaCl) (Zuhe Corporation, Nanjing, China).was used for overnight dialysis [25]. Then, 20 µL of each sample was detected by 12% SDS-PAGE. The remaining fusion protein was stored in a freezer at −80 °C.

### 2.8. Western Blot Analysis of the Bm_67459 Fusion Protein of B. multivorans WS-FJ9

The recombinant protein was subjected to SDS-PAGE electrophoresis and transferred to a PVDF membrane (0.45 mm). The membrane was kept in a blocking buffer containing 4% non-fat milk for 1.5 h, incubated with 1:1000 diluted anti-His antibody for 1 h at room temperature, and washed with a phosphate-buffered saline-Tween 20 (PBST) buffer 3 times (5 min each time). Then, after incubating with the secondary antibody for 0.5 h at room temperature, the membrane was washed 3 times with PBST washing buffer; the colour was developed with the Clarity Western ECL Substrate (BIO-RAD, Hercules, CA, USA) ultrasensitive luminescence colour developing solution and then photographed.

### 2.9. Antibacterial Effect of Phage Tail-like Protein Bm_67459 of B. multivorans WS-FJ9

The purified Bm_67459 protein was diluted to 10, 20, 30, 40, and 50 μg·mL^−1^, and 20 μL of protein solution at each concentration was dropped directly on the surface of pathogenic plugs. The control group was treated with heat-inactivated protein (incubated in boiling water for 45 min) and 1× PBS buffer solution. The samples were placed in a constant temperature incubator at 25 °C for 24 h, and the growth of pathogenic hyphae was observed using a Zeiss stereo microscope (Oberkochen, Germany). Each treatment was biologically repeated three times.

### 2.10. Statistical Analysis of the Data

Statistical analyses were carried out using Excel 2016 (Microsoft Corporation, Redmond, WA, USA) and SPSS software (ver. 23.0 IBM Corp., Armonk, NY, USA). Comparisons among treatments were analysed for significance using Duncan’s new multiple range test.

## 3. Results

### 3.1. Antagonistic Effect of B. multivorans WS-FJ9 on Forest Pathogens

**Figure 1:**
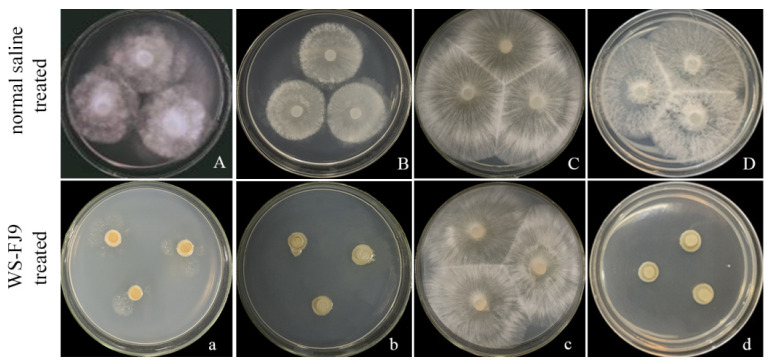
Detection of the activity of *Burkholderia multivorans* WS-FJ9 against plant pathogens. (**A**/**a**): *Phytophthora cinnamomi*, (**B**/**b**): *Phomopsis macrospore*, (**C**/**c**): *Rhizoctonia solani*, (**D**/**d**): *Sphaeropsis sapinea*.

Compared with the control treatment, the fermentation broth of the WS-FJ9 had strong inhibitory effects on *P. cinnamomi*, *P. macrospore,* and *S. sapinea,* but basically no inhibitory effect on *R. solani*. The surfaces of the pathogenic plugs of *P. cinnamomi*, *P. macrospore,* and *S. sapinea* treated with the WS-FJ9 strain were relatively moist and lacked mycelial growth (Figure 1), indicating that the WS-FJ9 strain had a strong inhibitory effect on the mycelial growth of the above three plant pathogenic strains. However, the mycelial growth on the surface of the *R. solani* plug treated with the WS-FJ9 was still vigorous and basically unchanged from that in the control group, indicating that the WS-FJ9 had no inhibitory effect on *R. solani* mycelia.

### 3.2. Effect of B. multivorans WS-FJ9 on Hyphae of Tree Pathogens by Electron Microscopy

**Figure 2:**
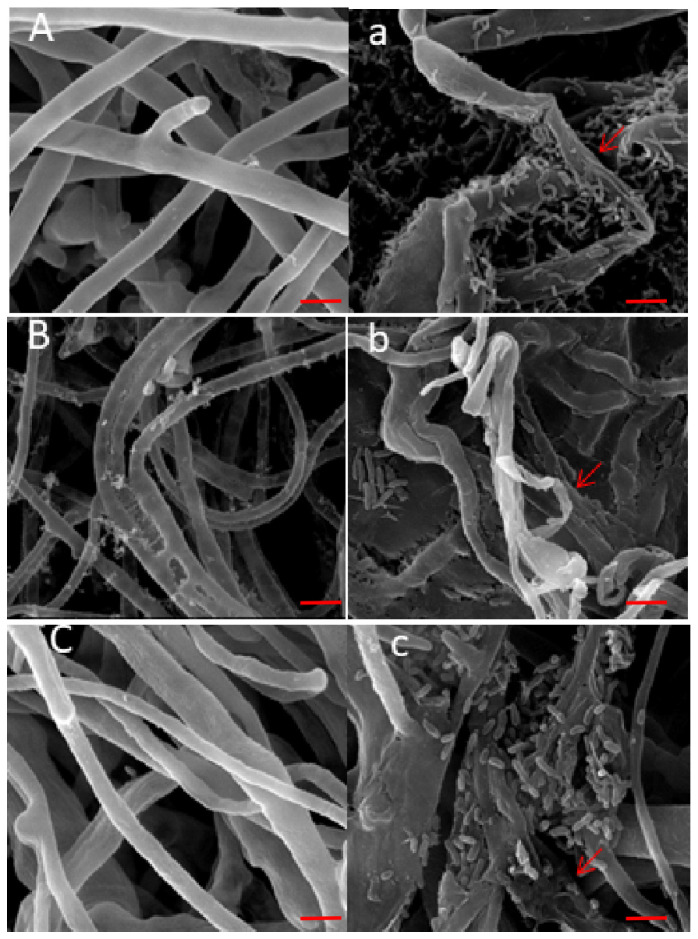
Mycelium of plant pathogens treated with normal saline and. *Burkholderia multivorans* WS-FJ9 observed by scanning electron microscopy. (**A**/**a**) *P*. *cinnamomi*; (**B**/**b**) *P*. *macrospore*; (**C**/**c**) *S*. *sapinea*; (**A**–**C**) Control: normal saline; (**a**–**c**) Treatment: WS-FJ9 suspension. (Note: The red arrow indicates damage to the mycelium). Scale bars = 50 µm.

The effect of WS-FJ9 on the hyphae of three pathogens was observed by scanning electron microscopy. After treatment with WS-FJ9, the mycelium of the pathogens appeared distorted, intertwined, twisted, folded, uneven in thickness, broken, shrivelled, and even split and dissolved on the surface of the mycelium (Figure 2).

### 3.3. Analysis and Cloning of Phage Tail-like Protein Genes from B. multivorans WS-FJ9

Analysing the genome of WS-FJ9 resulted in the annotation of 21 phage tail-like protein genes (Table 2). Most of these proteins existed in clusters in the genome, and their sizes varied greatly. The largest phage tail-like protein gene, contig67_457, was 2.64 kb, while the smallest was only 114 bp (contig67_458).

**Figure 3:**
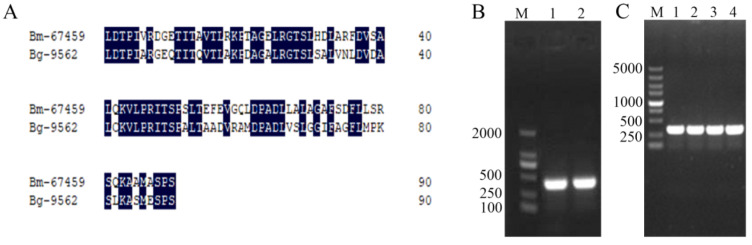
Amino acid sequence alignment between the *Bm_67459* and *Bg_9562* proteins and cloning of the *Bm_67459* gene. (**A**) Amino acid sequence alignment between the *Bm_67459* and *Bg_9562* proteins. (**B**) PCR amplification of the *Bm_67459* gene (M: DL2000 marker; 1,2: target gene *Bm_67459*). (**C**) PCR identification of *Bm_67459* colonies (M: DL5000 marker; 1~4: PCR identification of *Bm_67459* positive clones).

The 21 tail-like protein genes were analysed with BLASTN on the NCBI website, and the contig67_459 gene was 74% similar to the gene encoding the reported prophage tail protein Bg_9562 secreted by *B. gladioli* NGJ1 which has antifungal function [14]. Through multiple sequence alignment in DNAMAN, it was found that the amino acid sequence identity of contig67_459 and Bg_9562 protein was 62.64% (Figure 3A). We speculated that the protein encoded by the *contig67_459* gene had certain toxicity and renamed it the *Bm_67459* gene. Using the genomic DNA of WS-FJ9 as a template, the target gene *Bm_67459* was amplified by PCR. The result of agarose gel electrophoresis was shown in Figure 3B, with a bright band at 300 bp, consistent with the predicted size. The recovered and purified product was connected to the vector pMD^TM^19-T and transferred to *E. coli* DH5α. After colony PCR verification (Figure 3C), positive clones were sequenced, and the sequencing results were consistent with the sequence in the genome. The sequencing results were submitted to GenBank under the accession number MT742543.

### 3.4. Physicochemical Properties and Structural Analysis of the Phage Tail-like Protein Bm_67459 of B. multivorans WS-FJ9

**Figure 4:**
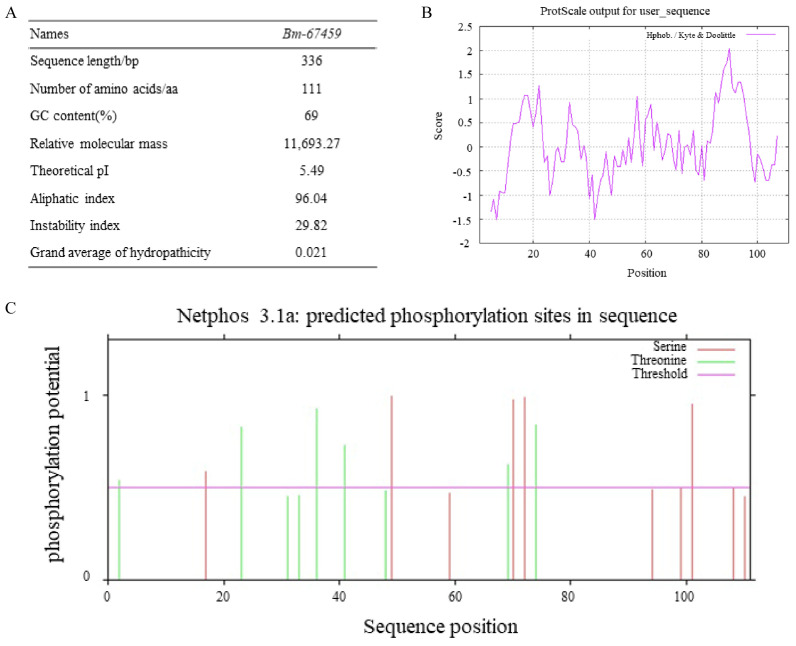
Bioinformatics analysis of the *Bm_67459* gene of *Burkholderia multivorans* WS-FJ9. (**A**) Physical and chemical parameters of the *Bm_67459* gene. (**B**) Hydrophobicity prediction for the Bm_67459 protein. (**C**) Phosphorylation site prediction for the Bm_67459 protein.

We analysed the physical and chemical properties of the Bm_67459 protein of the WS-FJ9. As shown in Figure 4A, the *Bm_67459* gene sequence was 336 bp in length and had a complete open reading frame (ORF). The GC content of the *Bm_67459* gene was high, at 69%. The relative molecular weight was approximately 11.69 kDa with 111 amino acids. The theoretical isoelectric point (pI) was 5.49, which indicated an acidic protein. The instability index was 29.82, indicating that the protein was stable (values below 40 indicate a stable protein). The predicted hydrophilicity of the Bm_67459 protein was shown in Figure 4B: with a hydrophilic index of 0.021, the protein was comfirmed hydrophilic. NetPhos 3.1 Server software analysis results showed that the Bm_67459 protein had 5 serine (Ser) and 6 threonine (Thr) residues, which might be protein kinase phosphorylation sites (Figure 4C). Based on the protein sequence, neither a signal peptide nor a transmembrane helix domain was present in the protein, and it could not be a receptor on the membrane or located on the membrane.

**Figure 5:**
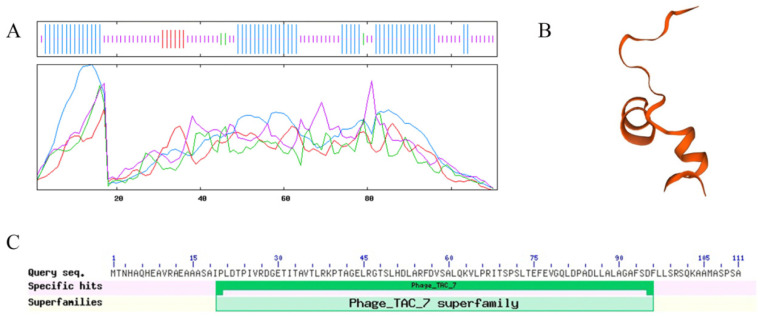
Secondary structure and tertiary structure prediction for the Bm_67459 protein. (**A**) Blue: alpha helix, red: extended strand, green: beta turn, purple: random coil. (**B**) Tertiary structure prediction for the Bm_67459 protein. (**C**) Prediction of the conserved domain of the Bm_67459 protein.

We analysed the secondary structure of the Bm_67459 protein of WS-FJ9 (Figure 5A). The Bm_67459 protein contained alpha helix, beta turn, extended strand, and random coil structures. The distribution of alpha helices in the secondary structure was high, at 45.95%. The second most common structure was random coil, accounting for 45.05% of the total secondary structure. Beta turned account for the lowest proportion, at only 2.70%. SWISS-MODEL software was used to generate a Bm_67459 protein model. The three-dimensional structures of the protein (Figure 5B) and the template Bm_67459 protein exhibited a protein sequence identity of 24.24% and overlapped for 44–76 amino acids. The three-dimensional structure of the Bm_67459 protein was mainly alpha helix and random coil with a small amount of beta turn, consistent with the secondary structure prediction results. The Bm_67459 protein was mostly one domain; it is located at amino acids 19–96 and belongs to the Phage_TAC_7 superfamily, indicating that the Bm_67459 protein belongs to this family (Figure 5C).

### 3.5. Differential Expression Analysis of the Phage Tail-like Protein Bm_67459 Gene of B. multivorans WS-FJ9

**Figure 6:**
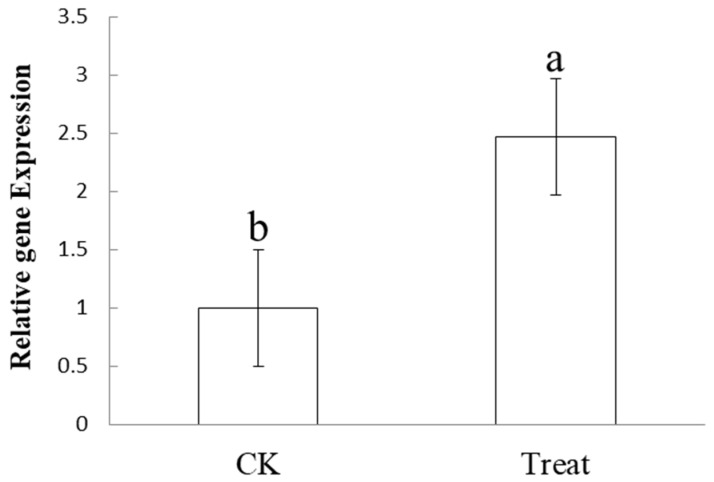
Differential expression analysis of the phage tail-like protein *Bm_67459* gene of *Burkholderia multivorans* WS-FJ9 upon *Phytophthora cinnamomi* contact. The significance was represented by a and b.

qRT-PCR was used to analyse the expression of the *Bm_67459* gene of WS-FJ9 before and after the interaction with *P. cinnamomi*. The relative expression of the *Bm_67459* gene was significantly upregulated by 2.5-fold after contact with *P. cinnamomi* (Figure 6). This result indicates that the *Bm_67459* gene was closely related to the antagonism of WS-FJ9 against *P. cinnamomi*.

### 3.6. Induced Expression and Purification of the Phage Tail-like Protein Bm_67459 of B. multivorans WS-FJ9

**Figure 7:**
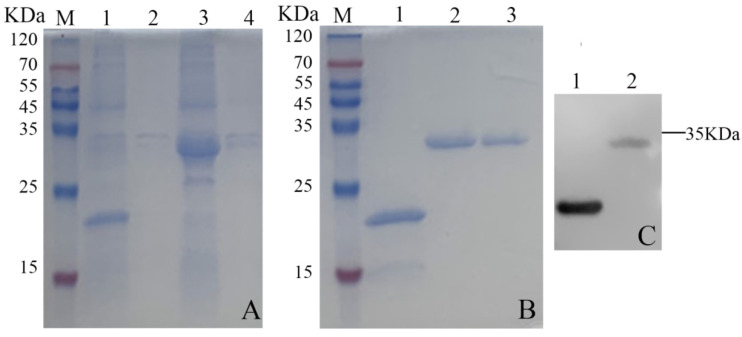
Prokaryotic expression, purification and Western blot analysis of the phage tail-like protein Bm_67459 of *B. multivorans* WS-FJ9. (**A**) Prokaryotic expression of the Bm_67459 protein; (M: protein molecular weight markers; 1: unpurified pET32a-carrying strain lysate supernatant; 2: unpurified pET-32a-carrying strain lysate supernatant; 3: unpurified pET32a-Bm_67459-carrying strain lysate supernatant; 4: unpurified pET-32a-Bm_67459-carrying strain lysate supernatant. (**B**) Purification of Bm_67459 protein (M: protein molecular weight markers; 1: purified lysate supernatant of pET32a; 2, 3-carrying strain: wash of Bm_67459 protein with 150 mmol·L^−1^ imidazole buffer). (**C**) Western blot detection of Bm_67459 protein; (1: purified pET32a-carrying strain lysate supernatant; 2: purified Bm_67459 protein).

The expression host bacteria were inoculated in a medium containing 0.5 mmol·L^−1^ IPTG, expression was induced at 37 °C for 4 h, and the empty vector prokaryotic expression product was used as a negative control. The crude recombinant protein was extracted and then detected by SDS-PAGE. There was an obvious protein band of approximately 32 kDa in the supernatant (Figure 7A, lane 3), which was consistent with the expected protein size, indicating that the fusion protein was successfully expressed in *E. coli* BL21. The Bm_67459 protein in the supernatant was purified by nickel ion affinity chromatography, and the eluted protein concentration was highest when the imidazole concentration was 100 mmol·L^−1^ or 150 mmol·L^−1^. The purified target protein was detected by SDS-PAGE, and a single protein band eluted at an imidazole concentration of 150 mmol·L^−1^ (Figure 7B, lanes 2 and 3). The recombinant protein was detected by Western blot, the purified Bm_67459 protein specifically reacted with an anti-6-His tag antibody, and the band size was consistent with the expected size (Figure 7C).

### 3.7. Antibacterial Activity of the Phage Tail-like Protein Bm_67459 of B. multivorans WS-FJ9

**Figure 8:**
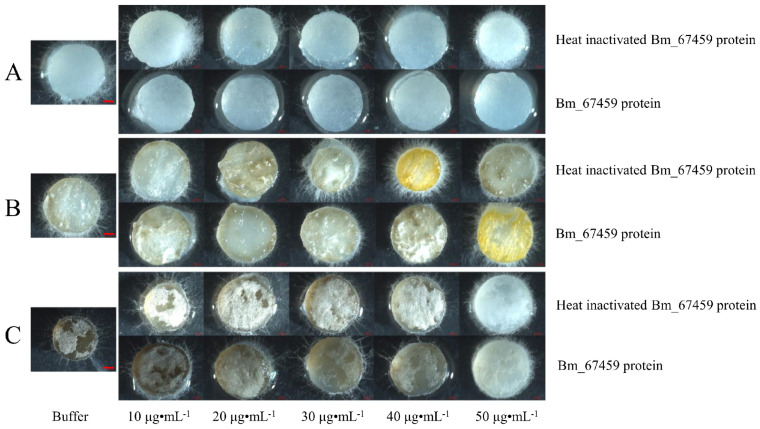
Antibacterial activity of the phage tail-like protein Bm_67459 of *B. multivorans* WS-FJ9. (**A**) *Phytophthora cinnamomi*; (**B**) *Phomopsis macrospore*; (**C**) *Sphaeropsis sapinea*. Scale bars = 50 µm.

To detect the bactericidal activity of the phage tail protein Bm_67459, the purified Bm_67459 protein was diluted to 10, 20, 30, 40, and 50 g·mL^−1^. Drops of the purified Bm_67459 protein were added to the surface of test plant pathogen plugs, and PBS buffer and heat-inactivated protein were added as a control. As shown in Figure 8, there was no inhibition of the three plant pathogens in the control group, and mycelium growth was relatively vigorous. The surface hyphae of *P. cinnamomi* after protein treatment were significantly less abundant than those of the control group, and even completely aseptic filaments grew. However, *P. macrospore* and *S. sapinea* treated with Bm_67459 protein did not show inhibition. This result shows that the Bm_67459 protein has a good specific inhibitory effect on *P. cinnamomi* and that it can inhibit mycelial growth at a concentration of 10 μg·mL^−1^.

## 4. Discussion

Plant growth-promoting rhizobacteria (PGPR) affect plant growth in both direct and indirect ways. PGPR directly promote plant growth by producing substances such as plant hormones, volatile compounds, and aminocyclopropane-1-carboxylic acid (ACC) deaminases, improving plant nutritional status (such as promoting the release of insoluble phosphorus, potassium, trace elements, and non-symbiotic nitrogen fixation) in plants [26]. On the other hand, PGPR contain various effective antibacterial factors, mostly secondary metabolites or substances in volatile gases, but the role of the secretion system of PGPR in antagonising plant pathogens is unclear. Our previous research has shown that the *B. multivorans* WS-FJ9 was focused mainly on the phosphorus solubilizing characteristics of the strain. For example, inoculation with WS-FJ9 could significantly improve the content of NL-895 poplar phosphorus, increase nitrate nitrogen and soluble protein contents, enhance nitrate reductase and root activities, and thus promote the growth of poplar [27]. This strain had strong adaptability and phosphate-solubilizing ability at low phosphorus levels, and the amount of organic phosphorus could be more than 140 mg·L^−1^ [23]. However, research on the antagonistic ability of WS-FJ9 against plant pathogens has not been reported. In this study, we found that the strain had strong antagonistic activity against both pathogenic fungi and oomycetes, for example, the fermentation medium of WS-FJ9 had strong antagonistic activity against *P. cinnamomi* (causes cedar root rot), *P. macrospore* (causes poplar canker) and *S. sapinea* (causes pine shoot blight), indicating that the WS-FJ9 strain had great biocontrol potential for preventing and controlling plant diseases.

Studies have shown that biocontrol bacteria mainly produce some cell wall-degrading enzymes (chitinase, glucanase, and protease), bacteriocins, hydrogen cyanide, and siderophores to antagonise plant pathogens [28]. In recent years, studies found that some bacteria could produce toxic effects on plant pathogens through the secretion of tailocins. Some bacteria could produce multiple tailocins simultaneously. For example, *Pseudomonas chlororaphis* produced two different R-tailocins, and they had different antibacterial spectra, which helped the strain compete with other rhizosphere-related bacteria [29]. *P. aeruginosa* PAO1 synthesised both R2 and F2 pyocins, the gene clusters of which were located next to each other. The R2 and F2 pyocins shared a common lysis cassette and regulatory system [30]. To further detect the active components of WS-FJ9 that antagonise plant pathogens, the whole genome of the WS-FJ9 strain was mined. The analysis revealed that WS-FJ9 had multiple phage tail protein gene clusters and 21 phage tail protein genes, indicating that the bacterium could synthesise multiple phage tail proteins. Comparing these 21 genes with NCBI database genes revealed that the *Bm_67459* gene was 74% similar to the reported *Bg_9562* gene [16], which has antifungal activity, and that the Bm_67459 protein had the main conserved domain of the phage tail protein family, indicating that the *Bm_67459* gene had potential antagonistic ability. Bioinformatics analysis showed that the Bm_67459 protein of the WS-FJ9 strain had no signal peptide or transmembrane structure, therefore we speculated that the protein is a non-classical protein [31].

Bacteriocins are ribosomally encoded peptides or proteins that exhibit high specificity [8,32]. Most tailocins exhibit narrow bactericidal spectra, displaying antibacterial activity against species of bacteria that are closely related to the producer [33]. Maltocin P28, an R-type tailocin produced by *Stenotrophomonas maltophilia* P28, is the only bacteriocin reported so far, and it killed only a fraction of the tested *S. maltophilia* strains [34]. Since bacteriocins can kill bacteria phylogenetically related to bacteriocin-producing strains without altering the remaining microflora, high specificity has become one of the advantages of bacteriocins. Therefore, bacteriocins are emerging as a promising alternative for reducing the application of agrochemicals in agriculture [35]. Príncipe investigated the efficacy of foliar application of tailocins to control bacterial spot disease in tomato caused by *Xanthomonas vesicatoria* Xcv Bv5-4a. The disease severity and incidence index were reduced by 44 and 36%, respectively [16]. To explore how the WS-FJ9 phage tail-like protein Bm_67459 functions in biological control against plant pathogens and to further develop Bm_67459 application in protein pesticide production, this study first used qRT-PCR to analyse the differential expression of the *Bm_67459* gene. It found that the gene was significantly upregulated during WS-FJ9 inhibition of *P. cinnamomi*, indicating that the protein played a certain role in the antagonism process.

When the purified Bm_67459 protein (10 μg·mL^−1^) was used to treat the oomycete *P. cinnamomea*, the growth of its hyphae could be significantly inhibited, but inhibitory effects on the plant pathogenic fungi *P. macrospore* and *S. sapinea* were not obvious, implying that the Bm_67459 protein had specificity against the oomycete *P. cinnamomi* and that the WS-FJ9 strain had potential for bio-controlling plant root rot caused by *P. cinnamomi*. *P. cinnamomi* is a soilborne pathogen that is widely distributed worldwide and harmful to trees and crops. It can cause root rot in trees and uses a wide range of plants, mainly *Camphora*, *Eucalyptus robusta*, *Persea lamericanal*, *Cedrus deodara,* and *Cupressus*, as hosts [36]. Maintaining high microbial activity in the soil could effectively control the occurrence of soilborne diseases. Some fungi and bacteria have antibiosis, competition, and mycoparasitism effects on *P. cinnamomi* [37]. A volatile organic compound produced by *Bacillus acidiceler* A8a inhibits the growth of *P. cinnamomi* by 76% [38]. *Rahnella aquatilis* JZ-GX1 ferrophilic fermentation broth also has a good inhibitory effect on *P. cinnamomi* [39]. Moreover, this study found that the WS-FJ9 strain can produce a tailocin and antagonise the growth of *P. cinnamomi*, which not only reveals the molecular mechanism of this strain’s bacteriostasis but also provides a resource for the biological control of *P. cinnamomi*. Bacterial strain resources lay the foundation for the development of new biological pesticides from microorganisms. Fermentation and cultivation of WS-FJ9 strain, collecting bacterial cells and placing them in suitable vector plasmids, could be used to produce bio-pesticides, and combined use with other pesticides can reduce the resistance of pathogenic bacteria and enhance drug efficacy. However, the environmental adaptability of the WS-FJ9 strain as a bio-pesticide still needs further consideration, and its effectiveness against other pathogenic fungi needs to be further improved. We performed only prokaryotic expression and purification of a tailocin from the WS-FJ9 strain. However, the antagonistic test of the fermentation broth suggested that some other antibacterial substances or mechanisms existed in the strain to exert antagonistic effects on the pathogenic fungi *P. macrospore* and *S. sapinea*. Esmaeel discovered multiple antibacterial genes and gene clusters in several genomes of Bcc strains [40]. Therefore, we need to further explore the genome of *B. multivorans* WS-FJ9 to identify more antibacterial genes and better analyse the antibacterial mechanism of this bacterium’s broad-spectrum activity.

## 5. Conclusions

The results showed that *B. multivorans* WS-FJ9 had a strong inhibitory effect on the growth of plant pathogenic fungi and oomycetes. The phage tail-like protein Bm_67459 of the WS-FJ9 strain is an important effector and has a specific antagonistic effect on *P. cinnamomi*. These findings provide insights into the antioomycete mechanism of *B. multivorans*. They also provide a new idea for the prevention and treatment of root rot disease and the study of new protein pesticide targets. In the future, we will further investigate the specific antibacterial substances of WS-FJ9 and test its inhibitory effect on *P. cinnamomi* in natural environments.

## Figures and Tables

**Table 1:** PCR primers used in the study.

Primers Name	Secquences (5′ to 3′)	Bases Number/bp	Funcion
Bm_67459-F	ATGACGAACCACGCTCAACACG	22	*Bm_67459* clone
Bm_67459-R	CGCAGACGGGGATGCCAT	18	
qBm_67459-F	ATGACGAACCACGCTCAACACG	22	*Bm_67459* clone
qBm_67459-R	GACCTCGAATTCCGTCAGCGA	21	
16s rDNA-F	GGGGAGTACGGTCGCAAGAT	20	Reference genes
16s rDNA-R	CATGTCAAGGGTAGGTAAGGTTT	23	

**Table 2:** Prophage tail-like protein genes of *Burkholderia multivorans* WS-FJ9.

Orf Name	Location	Protein Length	Predicted Function
contig67_457	9087–11,729	880	phage tail tape measure protein
contig67_458	11,726–11,842	38	GpE family phage tail protein
contig67_459	11,851–12,186	111	phage tail assembly protein
contig67_460	12,214–12,717	167	phage major tail tube protein
contig67_461	12,756–13,928	390	phage tail protein
contig67_462	13,982–14,587	201	phage tail protein
contig430_2941	25,664–26,125	153	phage tail protein
contig430_2944	29,164–30,354	396	phage tail protein
contig430_2945	30,773–31,993	406	phage tail protein
contig430_2946	32,388–33,608	406	phage tail protein
contig430_2947	33,679–34,128	149	phage tail protein
contig597_4008	6070–7005	311	phage tail protein
contig1014_6085	4909–5319	136	phage tail protein
contig1014_6087	6935–7384	149	phage tail protein
contig1014_6090	8856–9344	162	phage tail protein
contig1120_6584	1388–2494	368	phage tail protein
contig1120_6586	3847–6261	804	phage tail protein
contig1120_6588	6748–7125	125	phage tail protein
contig1120_6589	7155–8576	473	tail sheath protein
contig1519_7719	106–237	43	phage tail protein
contig1519_7720	351–806	151	phage tail protein

## Data Availability

The original contributions presented in the study are included in the article, further inquiries can be directed to the corresponding author.

## References

[1] Holland R.A., Crossthwaite A. (2024). Alkylsulfones: Novel chemical scaffolds targeting the vesicular acetylcholine transporter usher in a new generation of insecticides. Pest. Manag. Sci..

[2] Mani K., Vitenberg T., Khatib S., Opatovsky I. (2023). Effect of entomopathogenic fungus Beauveria bassiana on the growth characteristics and metabolism of black soldier fly larvae. Pestic. Biochem. Physiol..

[3] Haas D., Défago G. (2005). Biological control of soil-borne pathogens by fluorescent pseudomonads. Nat. Rev. Microbiol..

[4] Santhanam R., Menezes R.C., Grabe V., Li D., Baldwin I.T., Groten K. (2019). A suite of complementary biocontrol traits allows a native consortium of root-associated bacteria to protect their host plant from a fungal sudden-wilt disease. Mol. Ecol..

[5] Sugrue I., Ross R.P., Hill C. (2024). Bacteriocin diversity, function, discovery and application as antimicrobials. Nat. Rev. Microbiol..

[6] Kumariya R., Garsa A.K., Rajput Y.S., Sood S.K., Akhtar N., Patel S. (2019). Bacteriocins: Classification, synthesis, mechanism of action and resistance development in food spoilage causing bacteria. Microb. Pathog..

[7] Woudstra C., Sørensen A.N., Sørensen MC H., Brøndsted L. (2024). Strategies for developing phages into novel antimicrobial tailocins. Trends Microbiol..

[8] Ghequire M.G.K., Dillen Y.R., Lambrichts V., Proost P., Wattiez R., De Mo R. (2015). Different Ancestries of R Tailocins in Rhizospheric *Pseudomonas* Isolates. Genome Biol. Evol..

[9] Scholl D. (2017). Phage Tail-Like Bacteriocins. Annu. Rev. Virol..

[10] Nakayama K., Takashima K., Ishihara H., Shinomiya T., Kageyama M., Kanaya S., Ohnishi M., Murata T., Mori H., Hayashi T. (2000). The R-type pyocin of *Pseudomonas aeruginosa* is related to P2 phage, and the F-type is related to lambda phage. Mol. Microbiol..

[11] Canchaya C., Fournous G., Brüssow H. (2004). The impact of prophages on bacterial chromosomes. Mol. Microbiol..

[12] Salmond G.P.C., Fineran P.C. (2015). A century of the phage: Past, present and future. Nat. Rev. Microbiol..

[13] Godino A., Príncipe A., Fischer S. (2016). A ptsP deficiency in PGPR *Pseudomonas fluorescens* SF39a affects bacteriocin production and bacterial fitness in the wheat rhizosphere. Res. Microbiol..

[14] Fischer S., Godino A., Quesada J.M., Cordero P., Jofré E., Mori G., Espinosa-Urgel M. (2012). Characterization of a phage-like pyocin from the plant growth-promoting rhizobacterium *Pseudomonas fluorescens* SF4c. Microbiology.

[15] Fernandez M., Godino A., Príncipe A., Morales G.M., Fischer S. (2017). Effect of a *Pseudomonas fluorescens* tailocin against phytopathogenic *Xanthomonas* observed by atomic force microscopy. J. Biotechnol..

[16] Príncipe A., Fernandez M., Torasso M., Godino A., Fischer S. (2018). Effectiveness of tailocins produced by *Pseudomonas fluorescens* SF4c in controlling the bacterial-spot disease in tomatoes caused by *Xanthomonas vesicatoria*. Microbiol. Res..

[17] Yao G.W., Duarte I., Le T.T., Carmody L., LiPuma J.J., Young R., Gonzalez C.F. (2017). A Broad-Host-Range Tailocin from *Burkholderia cenocepacia*. Appl. Environ. Microb..

[18] Morse S.A., Jones B.V., Lysko P.G. (1980). Pyocin inhibition of *Neisseria gonorrhoeae*: Mechanism of action. Antimicrob. Agents Chemother..

[19] Swain D.M., Yadav S.K., Tyagi I., Kumar R., Kumar R., Ghosh S., Das J., Jha G. (2017). A prophage tail-like protein is deployed by *Burkholderia* bacteria to feed on fungi. Nat. Commun..

[20] Hou L. (2012). Studies on Screening of Efficient Phosphate-Solubilizing Bacteria in the Rhizosphere of Pine Trees and on Their Characteristics.

[21] Zeng Q., Wu X., Wang J. (2017). *Burkholderia multivorans* Phosphate Solubilization and Gene Expression of Phosphate-Solubilizing Bacterium WS-FJ9 under Different Levels of Soluble Phosphate. J. Microbiol. Biotechnol..

[22] Li G., Wu X., Ye J., Yang H. (2018). Characteristics of Organic Acid Secretion Associated with the Interaction between *Burkholderia multivorans* WS-FJ9 and Poplar Root System. Biomed. Res. Int..

[23] Liu Y., Wang Y., Kong W., Liu W., Xie X., Wu X. (2020). Identification, cloning and expression patterns of the genes related to phosphate solubilization in *Burkholderia multivorans* WS-FJ9 under different soluble phosphate levels. Amb Express.

[24] Livak K.J., Schmittgen T.D. (2001). Analysis of Relative Gene Expression Data Using Real-Time Quantitative PCR and the 2^−ΔΔCT^ Method. Methods.

[25] Zhang Y., Chen W., Li M., Yang L., Chen X. (2019). Cloning, phylogenetic research, and prokaryotic expression study of the metabolic detoxification gene EoGSTs1 in *Empoasca onukii* Matsuda. PEERJ.

[26] Beneduzi A., Ambrosini A., Passaglia L.M. (2012). Plant growth-promoting rhizobacteria (PGPR): Their potential as antagonists and biocontrol agents. Genet. Mol. Biol..

[27] Li G., Wu X., Ye J. (2014). Effects of *Burkholderia multivorans* WS-FJ9 on Nutrient Metabolism and Root Activity of Poplar. Acta Agric. Univ. Jiangxiensis.

[28] Bhattacharyya P.N., Jha D.K. (2011). Plant growth-promoting rhizobacteria (PGPR): Emergence in agriculture. World J. Microb. Biot..

[29] Dorosky R.J., Yu J.M., Pierson L.S., Pierson E.A. (2017). *Pseudomonas chlororaphis* Produces Two Distinct R-Tailocins That Contribute to Bacterial Competition in Biofilms and on Roots. Appl. Environ. Microb..

[30] Matsui H., Sano Y., Ishihara H., Shinomiya T. (1993). Regulation of pyocin genes in *Pseudomonas aeruginosa* by positive (prtN) and negative (prtR) regulatory genes. J. Bacteriol..

[31] Lloubes R., Bernadac A., Houot L., Pommier S. (2013). Non classical secretion systems. Res. Microbiol..

[32] Ahmad V., Khan M.S., Jamal Q.M.S., Alzohairy M.A., Al Karaawi M.A., Siddiqui M.U. (2017). Antimicrobial potential of bacteriocins: In therapy, agriculture and food preservation. Int. J. Antimicrob. Agents.

[33] Chen J., Zhu Y., Yin M., Xu Y., Liang X., Huang Y.P. (2019). Characterization of maltocin S16, a phage tail-like bacteriocin with antibacterial activity against *Stenotrophomonas maltophilia* and *Escherichia coli*. J. Appl. Microbiol..

[34] Liu J., Chen P., Zheng C.Y., Huang Y.P. (2013). Characterization of Maltocin P28, a Novel Phage Tail-Like Bacteriocin from *Stenotrophomonas maltophilia*. Appl. Environ. Microb..

[35] Mills S., Ross R.P., Hill C. (2017). Bacteriocins and bacteriophage; a narrow-minded approach to food and gut microbiology. Fems Microbiol. Rev..

[36] Hansen E. (2003). Phytophthora in North American forests. Sudden Oak Death Online Symposium.

[37] Wang M.S., Zhang S.H., Chen X.D. (2018). Research Progress on Biological Characteristics and Control Techniques of *Phytophthora cinnamomi*. J. Anhui Agric. Sci..

[38] Méndez-Bravo A., Cortazar-Murillo E.M., Guevara-Avendaño E., Ceballos-Luna O., Rodríguez-Haas B., Kiel-Martínez A.L., Hernández-Cristóbal O., Guerrero-Analco J.A., Reverchon F. (2018). Plant growth-promoting rhizobacteria associated with avocado display antagonistic activity against *Phytophthora cinnamomi* through volatile emissions. PLoS ONE.

[39] Kong W.L., Zhou M., Wu X.Q. (2019). Characteristics of siderophores production by *Rahnella aquatilis* JZ-GX1 and its antagonism against forest pathogens. Microbiol. China.

[40] Esmaeel Q., Pupin M., Kieu N.P., Chataigne G., Bechet M., Deravel J., Krier F., Hofte M., Jacques P., Leclere V. (2016). *Burkholderia* genome mining for nonribosomal peptide synthetases reveals a great potential for novel siderophores and lipopeptides synthesis. Microbiologyopen.

